# Effects of a Bioactive Vegetable-Enriched Diet on Autotaxin and Liver Fibrosis in MASLD with Evidence of Sex-Specific Responses: A Pilot Study

**DOI:** 10.3390/nu17233676

**Published:** 2025-11-24

**Authors:** Nicole Cerabino, Caterina Bonfiglio, Leonilde Bonfrate, Pasqua Letizia Pesole, Dolores Stabile, Endrit Shahini, Martina Di Chito, Giovanni De Pergola, Gianluigi Giannelli

**Affiliations:** 1 Center of Nutrition for the Research and the Care of Obesity and Metabolic Diseases, National Institute of Gastroenterology IRCCS “Saverio de Bellis”, Castellana Grotte, 70013 Bari, Italy; nicole.cerabino@irccsdebellis.it (N.C.); leonilde.bonfrate@irccsdebellis.it (L.B.); martina.dichito@irccsdebellis.it (M.D.C.);; 2 Unit of Data Science, National Institute of Gastroenterology IRCCS “Saverio de Bellis”, Castellana Grotte, 70013 Bari, Italy; 3 University San Raffaele, 00166 Rome, Italy; 4 Core Facility Biobank, National Institute of Gastroenterology IRCCS “Saverio de Bellis”, Castellana Grotte, 70013 Bari, Italy; letizia.pesole@irccsdebellis.it (P.L.P.);; 5 Department of Gastroenterology, National Institute of Gastroenterology IRCCS “Saverio de Bellis”, Castellana Grotte, 70013 Bari, Italy; endrit.shahini@irccsdebellis.it; 6 Scientific Direction, National Institute of Gastroenterology IRCCS “Saverio de Bellis”, Castellana Grotte, 70013 Bari, Italy; gianluigi.giannelli@irccsdebellis.it

**Keywords:** autotaxin, vegetable-enriched diet, MASLD, lysophosphatidic acid, liver fibrosis, Brassicaceae

## Abstract

**Background**: Metabolic dysfunction-associated steatotic liver disease (MASLD) is a frequent manifestation of obesity and other metabolic diseases. Autotaxin (ATX), an enzyme involved in the generation of lysophosphatidic acid (LPA), has recently emerged as a potential biomarker of metabolic inflammation and liver disease progression. Vegetable-based dietary interventions have been shown to reduce liver steatosis, but evidence of the impact of this dietary approach on ATX levels remains limited. **Objectives**: To evaluate the short-term effects of a bioactive vegetable-enriched diet from the *Brassicaceae* and *Asteraceae* families on serum ATX levels and liver-related parameters in individuals with obesity and MASLD, with a specific focus on sex differences. **Methods**: In this two-month pilot study, 44 obese adults (BMI > 30 kg/m^2^) underwent clinical and instrumental assessments at baseline (T0) and after the dietary intervention (T1). **Results**: After the intervention, serum ATX levels significantly decreased (from 206.3 ± 52.8 to 191.7 ± 45.7 ng/mL, *p* < 0.001), and there were improvements in metabolic parameters (BMI, waist circumference, blood pressure, fat mass, insulin, HOMA-IR, triglycerides, total and LDL cholesterol) and liver indices (CAP, ALT, AST, γGT). The multivariate GEE model confirmed a significant reduction in ATX, independent of age, sex, FFM, LPA, LSM, Hemoglobin A1c, and PAI-1 (β = −9.87, *p* < 0.001). When stratified by sex, women exhibited a more pronounced reduction in ATX levels (β = −12.24; *p* = 0.005) compared to men (β = −9.43; *p* = 0.014). **Conclusions**: A short-term, vegetable-enriched dietary intervention can significantly reduce serum ATX levels and improve metabolic and liver-related parameters in individuals with MASLD. Sex-specific analysis reveals a greater ATX-lowering effect in women, suggesting potential sex-based differences in ATX metabolism or dietary responsiveness. These findings suggest that ATX may serve as a modifiable biomarker responsive to nutritional intervention and a potential therapeutic target in metabolic liver disease.

## 1. Introduction

Metabolic dysfunction-associated steatotic liver disease (MASLD), previously classified under the broader umbrella of non-alcoholic fatty liver disease (NAFLD), has recently been redefined to emphasize its close relationship with systemic metabolic derangements [1]. The replacement of the old term NAFLD with MASLD highlights the growing awareness that hepatic steatosis does not occur alone but rather reflects a complex interplay of insulin resistance, dyslipidemia, visceral adiposity, and chronic low-grade inflammation [2,3]. MASLD is now recognized as the leading cause of chronic liver disease worldwide, affecting approximately one in three adults and up to 70–90% of individuals with obesity or type 2 diabetes mellitus [4,5].

Despite its increasing prevalence and its association with significant morbidity, including progression to metabolic steatohepatitis (MASH), advanced fibrosis, hepatocellular carcinoma, and cardiovascular disease, the pharmacological treatments for MASLD are focused on the optimal management of comorbidities (i.e., type 2 diabetes, dyslipidemia, hypertension, and sleep apnea) and liver damage. Clinical management is therefore predominantly based on lifestyle modifications, particularly dietary changes and weight loss [6]. The optimal dietary composition and specific food-based strategies capable of modulating liver fat accumulation and fibrosis are still areas under active investigation [7].

A growing body of evidence supports the beneficial role of vegetable-based dietary patterns in modulating hepatic lipid accumulation and inflammation, especially those containing abundant bioactive phytochemicals such as polyphenols, flavonoids, glucosinolates, and isothiocyanates [8]. These bioactive compounds are abundantly present in cruciferous vegetables, such as *Brassica oleracea* (e.g., cabbage, broccoli) and *Brassica rapa* (e.g., turnip greens, turnips), and exert antioxidant and anti-lipogenic effects by modulating various cellular pathways. A key mechanism involves the activation of AMP-activated protein kinase (AMPK), a central cellular energy sensor. Once activated, AMPK promotes catabolic processes that generate energy—such as fatty acid oxidation and glucose uptake—while simultaneously inhibiting anabolic processes such as lipid and protein synthesis. Through these actions, AMPK contributes to the sustainability of cellular energy homeostasis and may counteract pathological conditions associated with lipid accumulation, including obesity, hepatic steatosis, and metabolic syndrome [9].

Among the emerging molecular players implicated in the pathophysiology of MASLD, autotaxin (ATX) has garnered considerable interest due to its implication in the disease. ATX, also known as ectonucleotide pyrophosphatase/phosphodiesterase 2 (ENPP2), is a lysophospholipase D that catalyzes the conversion of lysophosphatidylcholine (LPC) to lysophosphatidic acid (LPA)—a potent bioactive lipid involved in several biological processes including fibrosis, adipogenesis, inflammation, and carcinogenesis [10]. The ATX–LPA signaling axis is upregulated in metabolic conditions such as obesity and insulin resistance, and clinical studies have demonstrated elevated serum ATX levels in patients with chronic liver disease, correlated with the severity of steatosis, fibrosis, and systemic inflammation [11].

ATX has also been proposed as a mechanistic link between adipose tissue dysfunction and liver disease. In obese individuals, hypertrophic and inflamed adipocytes overexpress ATX, resulting in elevated circulating LPA levels that promote hepatic stellate cell activation, fibrogenesis, and immune cell recruitment [12]. Moreover, LPA modulates hepatic glucose and lipid metabolism via its relationship with LPA receptors (LPA1–6), further exacerbating insulin resistance and hepatic lipid depots. These findings suggest that ATX may serve not only as a biomarker of liver disease progression but also as a potential target for therapeutic intervention [13].

Although several studies have investigated the association between ATX and advanced liver disease, few have examined the impact of dietary interventions on ATX levels in the early stages of MASLD. In particular, the effect of short-term, vegetable-enriched dietary modifications, especially those incorporating bioactive-rich vegetables from the Brassicaceae family, on ATX concentrations has yet to be elucidated.

Moreover, recent evidence suggests that sex differences may influence both the progression of MASLD and the expression of key molecular mediators such as ATX. Obese women often exhibit distinct patterns of adipose tissue distribution and inflammation, potentially affecting ATX secretion and LPA signaling [14]. Hormonal regulation, particularly estrogen-related pathways, may also modulate ATX activity and hepatic responses to dietary changes, highlighting the importance of sex-stratified analyses in nutritional interventions.

In this context, given the lack of preliminary interventional data, we designed a pilot study to test the feasibility and biological plausibility of a vegetable-enriched diet on ATX modulation in MASLD. This pilot study aimed to assess whether substituting a single daily portion of starchy carbohydrates with a 200 g serving of selected vegetables (including *Brassica rapa* var. *cymosa*, *Brassica oleracea* var. *sabellica*, *Cichorium intybus*, and *Sinapis arvensis*) could modulate circulating ATX levels, liver fibrosis, and metabolic parameters in individuals with obesity and MASLD. These specific vegetables were selected based on their phytochemical profile and documented hepatoprotective properties in preclinical studies [15]. Additionally, we explored sex-related differences in the response to the dietary intervention, focusing on whether changes in ATX and liver-related outcomes differ between men and women. We investigated the correlation between changes in ATX levels and liver stiffness measurements (LSM), based on the hypothesis that decreasing ATX levels could reflect concurrent improvements in hepatic fat content and fibrosis risk.

This pilot study is one of the first attempts to evaluate the modulation of ATX levels following a feasible food-based intervention. By exploring the interaction between vegetable-based nutrition and the ATX–LPA axis, together with sex-related metabolic responses, we aim to contribute to the growing field of precision nutrition in metabolic liver disease.

## 2. Materials and Methods

### 2.1. Study Design and Population

This is a two-month prospective interventional pilot study conducted by the Clinical Nutrition Research Centre for Obesity and Metabolic Diseases at the Saverio de Bellis National Institute of Gastroenterology in Castellana Grotte (Bari, Italy).

A pilot study is a small-scale investigation designed to assess the practicality and feasibility of methods before a more comprehensive study [16]. Eligible participants were adults aged 18 to 65 years with a body mass index (BMI) over 30 kg/m^2^ and not taking any current medications. Exclusion criteria included diagnosed or newly discovered diabetes mellitus, cardiovascular disease, respiratory failure, severe gastrointestinal disorders, irritable bowel syndrome, chronic kidney disease (e.g., estimated glomerular filtration rate < 60 mL/min/1.73 m^2^), psychiatric illnesses, pregnancy or breastfeeding, eating disorders, chronic liver disease of non-metabolic origin, alcohol consumption exceeding 30 g/day for men or 20 g/day for women, substance abuse, infectious diseases, acute illnesses that could influence inflammatory markers, rare metabolic diseases, or mitochondrial fatty acid oxidation disorders.

Obese participants underwent anthropometric measurements and bioimpedance analysis (BIA), clinical history assessment, and laboratory evaluations (hematological and biochemical parameters). Physical activity levels were assessed using the International Physical Activity Questionnaire (IPAQ) [17] and the adherence to the Mediterranean diet was evaluated through the PREDIMED questionnaire [18]. Smoking status was also recorded.

The pilot study protocol received approval from the local Medical Ethics Committee (Protocol no. 681 dated 15 November 2023) and was conducted in compliance with the ethical standards of the 1964 Declaration of Helsinki. All subjects provided written informed consent before enrollment. Recruitment was conducted between May 2023 and December 2024. Two clinical visits were scheduled: at baseline (T0) and after two months (T1). At both visits, data collection included fasting blood samples, anthropometric evaluation, and instrumental assessments (BIA and FibroScan).

**Figure 1:**
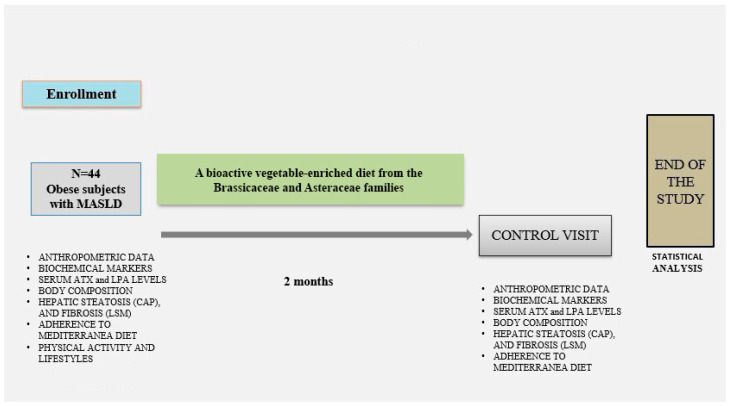
The study design was a two-month prospective interventional pilot study. After enrollment, subjects were instructed to replace one daily portion of starchy carbohydrates with 200 g of selected vegetables (*Brassica rapa*, *Brassica oleracea*, *Cichorium intybus*, *Sinapis arvensis*). At baseline, the subjects underwent clinical history assessment, and evaluation of serum ATX and LPA levels, biochemical markers, body composition, hepatic steatosis (CAP), and fibrosis (LSM), physical activity levels and adherence to the Mediterranean diet through the PREDIMED questionnaire. At the end of the nutritional approach, subjects underwent assessment of serum ATX and LPA levels, biochemical markers, body composition, hepatic steatosis (CAP), and fibrosis (LSM), as well as adherence to the Mediterranean diet through the PREDIMED questionnaire.

Participants were instructed to fill out a three-day self-reported food diary, including two weekdays and one weekend day. Based on their usual dietary habits, they were asked to replace one daily portion of carbohydrates (e.g., bread, pasta, or potatoes) with a 200 g serving of vegetables. No further dietary or lifestyle modifications were recommended. The vegetables included four species from the Brassicaceae family: Brassica rapa var. cymosa, Cichorium intybus, Brassica oleracea L. var. sabellica, and Sinapis arvensis var. orientalis [19]. These vegetables are recognized as sources of bioactive compounds such as isothiocyanates and phenolic substances, which have shown antioxidant and anti-lipogenic effects in preclinical models of non-alcoholic fatty liver disease (NAFLD) [20] (see Figure 1).

### 2.2. MASLD Assessment

FibroScan is a non-invasive, cost-effective, and validated tool for the assessment of hepatic steatosis and fibrosis in at-risk individuals [21]. Although liver biopsy remains the gold standard for diagnosing steatosis, fibrosis, and hepatic inflammation, ultrasound-based elastography using FibroScan provides a painless and comprehensive assessment of liver health and is recommended as a first-line diagnostic approach [22].

Hepatic fat accumulation was assessed using vibration-controlled transient elastography (VCTE) in combination with the Controlled Attenuation Parameter (CAP) at a frequency of 3.5 MHz. The rule-in cut-off for the steatosis definition was CAP > 275 dB/m [23]. Liver stiffness measurement (LSM) was used to assess hepatic fibrosis, with cutoff values of 8 kPa and 12 kPa indicating fibrosis and advanced fibrosis (stage 3), respectively. No subject showed LSM values consistent with advanced fibrosis or cirrhosis (LSM ≥ 12 kPa).

### 2.3. Anthropometric Parameters

All measurements were obtained under standardized conditions, with participants in a fasting state, barefoot, wearing light clothing, and with an empty bladder. Height and body weight were recorded using standardized tools, and the BMI was calculated and classified according to the criteria established by the National Institutes of Health and WHO criteria: underweight (BMI < 18.5 kg/m^2^); normal weight (BMI ≥ 18.5–24.9 kg/m^2^); overweight (BMI ≥ 25.0–29.9 kg/m^2^); obese (BMI ≥ 30 kg/m^2^). Waist circumference was assessed at the midpoint between the lower margin of the last palpable rib and the superior border of the iliac crest, using a horizontal measuring plane. Values ≥ 94 cm in men and ≥80 cm in women were used as thresholds to define abdominal obesity and elevated cardiometabolic risk [24]. Systolic and diastolic blood pressure (SBP and DBP) was recorded three times with participants seated and at rest, using an automatic monitor (OMRON M6).

### 2.4. Bioelectrical Impedance Analysis (BIA)

Body composition was assessed using a single-frequency bioimpedance analyzer (BIA-101, 50 kHz; Akern Bioresearch, Florence, Italy). In accordance with ESPEN recommendations [25], participants were evaluated in the supine position, with legs slightly apart. They were instructed to avoid eating, drinking alcohol, or engaging in physical activity for at least 12 h before the test. After disinfecting the skin, injector electrodes were placed on the dorsal side of the right hand and the upper surface of the right foot, while sensor electrodes were positioned on the right wrist and between the medial and lateral malleoli of the right ankle [26]. Resistance (RZ) and reactance (Xc) were measured, and parameters such as fat mass (FM), fat-free mass (FFM), total body water (TBW), and extracellular water (ECW) were computed using dedicated software algorithms.

### 2.5. Biochemical Analyses

Fasting blood samples were collected in the early morning. Blood cell count was evaluated by fluorescence flow cytometry using the automatic hematology analyzer Sysmex XT-1000 (Dasit, Cornaredo, Milano, Italy). Serum was analyzed for fasting serum glucose (FSG), fasting insulin, triglycerides, total cholesterol, LDL-C, HDL-C, AST, ALT, GGT, uric acid, creatinine, high-sensitivity C-reactive protein, thyroid hormones, and 25-hydroxyvitamin D. Analyses were carried out using the COBAS 8000 autoanalyzer (ROCHE Diagnostic SPA, Monza, Italy).

Glycated hemoglobin (HbA1c) was measured with the Capillarys 3 OCTA capillary electrophoresis system (Sebia Italia S.r.l., Bagno a Ripoli, Florence, Italy).

Insulin resistance was calculated using the Homeostasis Model Assessment of Insulin Resistance (HOMA-IR), according to the following equation [27]:

HOMA-IR = FSG (mg/dL) × fasting insulin (μIU/mL)/405

Serum autotaxin levels were quantified using an ELISA assay, following the manufacturer’s protocol (Human ENPP-2/Autotaxin Immunoassay ELISA kit, R&D SYSTEM, Bio-techne, Minneapolis, MN, USA). The minimum detectable concentration of human ENPP-2 ranged between 0.055 and 0.157 ng/mL.

Lysophosphatidic acid (LPA) levels in serum were determined using an ELISA kit in accordance with the manufacturer’s instructions (Human Lysophosphatidic Acid ELISA kit, Cusabio, Houston, TX, USA). The assay sensitivity was <3.9 ng/mL.

### 2.6. Variables of Exposure and Confounders

The exposure variable was a vegetable-enriched diet intervention. Seven confounding parameters—sex, age (<50 vs. ≥50 years), LSM, FFM, LPA, PAI-1, and Hemoglobin A1c—were considered in the final multivariate statistical model to adjust the association estimates between the effects of the two-month dietary intervention and circulating ATX levels.

When selecting variables as confounders for the model, those already included in the definition of MASLD, such as BMI, waist circumference, HDL cholesterol, triglycerides, fasting glucose, and hypertension, were excluded.

### 2.7. Statistical Methods

We performed statistical analysis of baseline variables, expressed as Mean ± Standard Deviation (SD), median and range for continuous variables. We used the Wilcoxon rank sum test for paired data, with continuous variables to compare two groups, and the χ^2^ test to evaluate differences in categorical variables. Statistical significance was determined with 95% Confidence Intervals (CI) for *p*-values of 0.05 or less. Additionally, a retrospective power analysis was conducted to demonstrate that our sample was adequate, and hence the results were reliable.

The correlation coefficient, along with the relative *p*-value, between the differences before and after the dietary intervention for ATX and LSM, was calculated and displayed in the linear prediction plot [28].

A Generalised Estimating Equation (GEE) [29] was used to estimate the longitudinal trajectories of ATX (pre- and post-vegetable-enriched diet).

GEE models help estimate mean changes in biomarker values while adjusting for covariates in biomedical investigations because they allow for correlations of response data (i.e., repeated measurements in each subject). Due to the non-normal distribution of the outcome variables, a gamma distribution (link identity) was used to model the response, and an unstructured correlation matrix was applied to the data.

Initially, confounding variables were selected from the existing literature. Then, the minimum absolute reduction and selection (LASSO) was adopted to reduce the number of candidate predictors and select those most useful for the model construction [30].

In addition, the variance inflation factor (VIF) was also evaluated to check multicollinearity, and confounders with VIF > 5 were discarded [31].

The use of many variables can increase the risk of multicollinearity, in which predictor variables are highly correlated, making it difficult to isolate the true effect of each variable. The rule of having at least 10–15 observations for each independent variable is a general guideline for statistical modeling, particularly regression, to ensure reliable results [32].

Time of Diet Intervention was considered the exposure variable, while Sex, Age (<50 vs. ≥50 years), LSM, LPA, PAI1, and Hemoglobin A1c were all considered covariates.

Five models were developed to estimate how diet affects ATX: Model a is univariate; Model b adjusts for sex and age; Model c adjusts for sex, age (<50 vs. >50 years), LSM, LPA, FFM, PAI1, and Hemoglobin A1c; Model d is univariate and stratified by sex; and Model e is multivariate, adjusted for age (<50 or >50 years), LSM, LPA, FFM, PAI1, Hemoglobin A1c, and stratified by sex.

Stata statistical software, version 19.0 (StataCorp, 2025, 4905 Lakeway Drive, College Station, TX, USA), was used for statistical analysis.

## 3. Results

As a pilot trial, the analysis focused on within-subject changes and exploratory sex-stratified patterns rather than definitive inter-group comparisons.

The sample included an equal proportion of males and females (all sample = 44; males = 22, females = 22; M:F ratio = 1:1). Nine drop-outs were recorded. The mean age of women was 45.15 years (11.46), while for men it was 47.71 years (9.49). All participants were classified along a continuum degree of obesity. At baseline, women displayed a BMI of 36.6 (4.88), which decreased to 34.74 (4.55) after two months. The majority of subjects did not report a smoking habit. The adherence to the Mediterranean Diet was low in 5 (11%), moderate in 28 (64%), and high in 11 (25%) of subjects. Physical activity was <30 min in 8 (20%), >30 min in 33 (80%) of subjects. According to educational levels, secondary school was reported in 11 (27%), high school in 22 (54%), and University in 8 (20%) of subjects.

As reported in Table 1, there were no differences between sexes regarding the percentage of subjects with a smoking habit, physical activity levels and education level. Table 1 presents the baseline demographic and lifestyle features by sex.

Table 2 reports the overall sample data before and after the implementation of the vegetable-enriched dietary intervention. Both CAP (the Fibroscan parameter for steatosis) and liver stiffness (the Fibroscan parameter for fibrosis) were lower following the diet. After the dietary intervention, there was a general improvement in all measured values, particularly for ATX, Systolic and Diastolic Blood Pressure, BMI, waist circumference, fat mass, insulin, Homa IR, glycated haemoglobin, triglycerides, total and LDL cholesterol, ALT, AST, and GGT, all of which were significantly reduced.

The post-diet improvements reported in Table 2 are also apparent in Table 3, where the data are broken down by sex. The decrease in ATX is greater among women than men (−17.99 ng/mL versus −11.2 ng/mL, respectively). The reduction in LSM following the diet in women is statistically significant (*p*-value = 0.0356). Changes in eating habits are evident in both sexes, as shown by data from the PREDIMED questionnaire completed before and after the dietary intervention. The most notable differences between men and women due to dietary treatment are seen in CAP (−27 dB/m for women and −43 dB/m for men), LSM (−1.29 kPa for women and −0.26 kPa for men), and triglycerides (−38 mg/dL for women and only −6 mg/dL for men).

Table 4 presents the differences between males and females before and after the diet. Significant differences after the diet are observed in DBP (*p*-value < 0.001) and triglycerides (*p*-value 0.009), which were not present before the diet. For CAP (*p*-value 0.004), ALT (*p*-value 0.006), γGT (*p*-value = 0.039), FT3 (*p*-value 0.009), and FT4 (*p*-value 0.005), sex differences are statistically significant but only evident in the pre-diet phase. Statistically significant sex differences before and after the diet are confirmed for ATX, waist circumference, free fat mass, body cell mass, insulin, HOMA IR, HDL, Uric Acid, Creatinine, WBC, and RBC. No sex differences are observed for the other variables before or after the diet.

**Figure 2:**
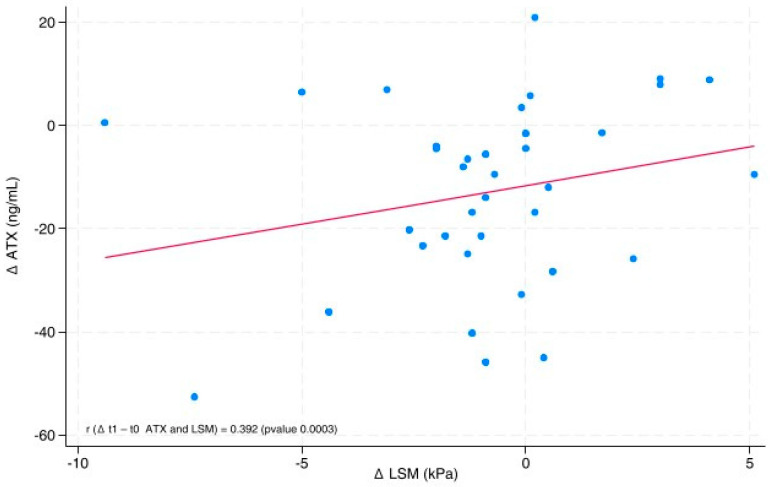
Linear and scatter plot between ΔATX and ΔLSM. r: correlation coefficient; ATX: Autotaxin; LSM: Liver Stiffness Measurement.

The line graph (Figure 2) shows the directly proportional relationship between ∆ATX (ATX post-diet—ATX pre-diet) and ∆LSM (LSM post-diet—LSM pre-diet), with a correlation coefficient (r) of 0.392 (*p*-value 0.0003). We also used a scatter plot to represent the values of ∆ATX and ∆LSM. The position of each point on the horizontal and vertical axes indicates the values of a single data point.

Table 5 presents the results of three regression analysis models constructed with GEE to examine the relationship between the vegetable-enriched diet and ATX in patients with MASLD. A statistically significant association with the effect of diet was found in model a (β = −13.80; *p*-value < 0.001). Since model *a* is univariate, the beta regression coefficient, which estimates the mean change in ATX before and after follow-up, only considers the effect of diet and ignores all potential confounding factors. When adjusting for sex and age, as in model *b*, there is a minor reduction in ATX before and after diet, indicated by the coefficient value: β = −12.44 (*p*-value < 0.001). This suggests that age and sex influence the change in ATX before and after the diet.

Analysing the multivariate *c*-model, the estimated effect of diet on ATX, represented by the beta coefficient, is −9.87 with a *p*-value < 0.001. In contrast, the effect of LSM on ATX is β = 2.25 (*p*-value = 0.009).

In other words, ATX decreased significantly by 9.87 ng/mL after the vegetable-based diet, and each unit decrease in LSM was associated with a statistically significant 2.25 ng/mL reduction in ATX, controlling for sex, age (<50 vs. ≥50 years), LPA, FFM, PAI1, and Hemoglobin A1c. Thus, the reduction in ATX observed at the end of the dietary intervention is smaller than in previous regression models *a* and *b*, which could be due to the adjustments made in model *c*, which includes sex, age, LPA, FFM, Hemoglobin A1c, PAI1, and LSM. (See Table 5)

Table 6 presents the results of regression analyses conducted using the GEE model to illustrate the associations between the effect of a vegetable-enriched diet and ATX in patients with MASLD, classified by sex. In the univariate model, a statistically significant association was observed for the effect of diet in both women and men (β = −17.96; *p*-value < 0.001, β = −9.68, *p*-value = 0.002, respectively). A greater decrease in ATX was observed in women than in men.

Multivariate analysis indicates a smaller decrease in ATX in women (β = −12.24, *p* = 0.005), due to the inclusion of the covariates previously described in model *c.* Furthermore, the effect of LSM on ATX was β = 2.18 (*p* = 0.049).

In males, the difference in ATX between the univariate and multivariate models is almost negligible (β = −9.68, *p*-value = 0.002 and β = −9.43, *p*-value = 0.014, respectively), and LSM is not statistically significant (β = 2.61, *p*-value = 0.078).

## 4. Discussion

As a preliminary, small-sample pilot study, our findings provide early evidence supporting the beneficial role of a simple and well-tolerated dietary modification—replacing one daily serving of starchy carbohydrates with 200 g of vegetables from the Brassicaceae and Asteraceae families—in significantly reducing serum autotaxin (ATX) levels in individuals with obesity and MASLD. This reduction was accompanied by clinically relevant improvements in several metabolic (BMI, waist, blood pressure, insulin, HOMA-IR, triglycerides, total and LDL cholesterol), hepatic (CAP, ALT, AST, γGT), and inflammatory parameters, suggesting a broad beneficial impact on metabolic and liver health.

The most striking result is the significant decrease in ATX levels, which remained evident even after adjusting for potential confounders (age, sex, FFM, LPA, LSM, hemoglobin A1c, and PAI-1) in the multivariate model. This supports the hypothesis that ATX is a modifiable biomarker responsive to nutritional intervention and a potential therapeutic target in metabolic liver disease.

A more detailed analysis of the data highlighted potential sex-related trends in the biological response to the dietary intervention. Both sexes exhibited significant improvements in metabolic, hepatic, and inflammatory parameters following the intervention, even though these improvements were often not coincident. This finding confirms the overall effectiveness of the intervention for the entire study population. In contrast, the decrease in serum ATX levels was evident in both sexes, with a significantly greater reduction in females. This finding may suggest a possible sex-specific modulation of the ATX–LPA axis, although further studies with larger and stratified cohorts are needed to confirm this hypothesis [33]. From a biological perspective, estrogens have been reported to negatively regulate ATX expression, potentially contributing to the greater reduction observed in females. Moreover, differences in adipose tissue distribution—predominantly subcutaneous in women and predominantly visceral in men—may influence inflammatory signaling and adipokine secretion, thereby modulating ATX production [34,35]. Since ATX is secreted by both adipocytes and damaged hepatocytes, these sex-specific tissue patterns could affect circulating levels.

In women, the reduction in ATX was linked to a clinically meaningful improvement in liver stiffness (LSM: from 7.92 to 6.63 kPa; *p* = 0.036) and significant decreases in triglycerides and total cholesterol, along with a reduction in waist circumference. In men, the reduction in ATX was significant but less marked, with the most notable benefits involving liver function and glucose metabolism. Specifically, a significant decrease in transaminases and γGT was observed, along with improved insulin resistance. Additionally, the decrease in circulating white blood cells suggests a favorable effect on systemic inflammation, consistent with the greater contribution of visceral adipose tissue in men [36]. Overall, the data demonstrate that, although the vegetable-enriched diet led to clinical benefits in both sexes, the response patterns differed. In women, improvements were mainly related to liver fibrosis and lipid profile, whereas in men, they concerned hepatocellular injury and insulin resistance. While these differences were not the primary focus of the pilot study, they highlight the role of sex dimorphism in influencing the nutritional impact on MASLD and underscore the importance of including sex-based analyses in future clinical trials.

### 4.1. Clinical Implications and Strengths

One of the main strengths of this pilot study is the simplicity and real-life applicability of the intervention. Participants were not subjected to complex or restrictive diets but simply replaced one carbohydrate portion with bioactive-rich vegetables known for their antioxidant, anti-inflammatory, and anti-fibrotic effects [15,37]. In particular, the study by Iwadare et al. [37] demonstrated that serum autotaxin levels represent a prognostic indicator of liver-related events in patients with NAFLD, supporting the clinical value of this biomarker. ATX emerges as a promising biomarker for monitoring MASLD progression and treatment response. Its sensitivity to dietary changes, independent of clinical and non-clinical confounding factors, reinforces its potential role in precision nutrition for metabolic liver disease [37,38]. Supporting this, the research conducted by Liu et al. [37] demonstrated that pharmacological inhibition of autotaxin exerts anti-fibrotic effects at the hepatic level, which also opens intriguing possibilities for nutritional integration.

The sex-specific outcomes observed further underline the importance of adopting a sex-based perspective in the management of MASLD. Tailoring dietary interventions based on sex may enhance therapeutic efficacy and ensure a more individualized approach to care.

### 4.2. Limitations of the Study

Despite its methodological quality, this study has some limitations. Firstly, the fairly short duration of the intervention may have undervalued slower metabolic benefits and impacted the glycemic profile and liver fibrosis. Further studies with extended follow-up will be needed to estimate the sustainability and clinical applicability of the observed changes. Secondly, the relatively small sample size may have reduced the statistical power.

In addition, the study design did not include a control group, despite the aim of this study being to evaluate a feasible and sustainable nutritional approach model. This pilot study was designed to evaluate the biological variability and feasibility of a simple, diet-based intervention in subjects with MASLD. Patients were instructed not to alter their lifestyle in terms of physical activity, since this was not an endpoint of the study. Furthermore, physical activity and PGWBI were assessed only at baseline to describe the cohort, as the relatively short duration of the intervention (two months) did not allow us to predict significant changes in these parameters.

We acknowledge that changes in body composition may have influenced ATX levels. In our multivariate model, we included fat-free mass (FFM) as a covariate, and the reduction in ATX remained significant, suggesting that the observed effect is not entirely explained by such changes. However, we acknowledge that fat loss may have contributed in part to the result.

Another limitation of the study is the absence of a detailed phytochemical analysis of the vegetables consumed and the possible inter-individual variability in their intake. This diversity may have incompletely impacted the observed effect, making it more difficult to attribute the benefit to a specific bioactive emulsion. Future studies will need to include the precise quantification of ingested phytocompounds to better define the observed associations.

Given the promising results obtained with this healthy dietary model, it would be intriguing to estimate in future studies the effects of other beneficial dietary patterns on the same molecules that were included in the present study.

We recognize that the accuracy of FibroScan may be reduced in obese individuals due to the thickness of subcutaneous adipose tissue. However, we utilized the XL probe in accordance with ESPEN recommendations, which enhances measurement accuracy in this population.

Additionally, while analyses highlighted intriguing sex-related differences, the study was not originally powered or stratified to explore sex effects in depth.

Future studies should incorporate stratified analyses by sex and consider hormonal status, body composition, and metabolic phenotype to tailor dietary strategies more precisely. Integrating sex-specific insights could pave the way for personalized nutritional approaches in the management of MASLD and related metabolic disorders.

These findings should therefore be interpreted as hypothesis-generating and serve as a rationale for larger randomized controlled trials.

Overall, these limitations are consistent with the exploratory nature of pilot studies and do not detract from the feasibility and biological trends observed.

## 5. Conclusions

This pilot study provides new evidence that a short-term, vegetable-enriched dietary intervention—based on the simple substitution of one daily portion of starchy carbohydrates with 200 g of vegetables from the Brassicaceae and Asteraceae families—can significantly reduce serum autotaxin (ATX) levels in individuals with obesity and MASLD. This reduction was accompanied by improvements in metabolic, hepatic, and inflammatory parameters, highlighting the potential of ATX as a modifiable biomarker [37] and a nutritional therapeutic target in metabolic liver disease.

The intervention was practical, well-tolerated, and required minimal changes to the participants’ usual dietary habits, suggesting a strong potential for implementation in clinical and public health settings.

Most importantly, the study revealed significant sex-based differences in the response to the dietary intervention, both in terms of ATX modulation and in broader metabolic outcomes. These findings underscore the importance of incorporating sex-based analyses into future research and support the development of personalized dietary strategies to improve liver and metabolic health in both men and women.

In this context, experimental evidence has shown that adipose-specific deletion of autotaxin reduces circulating lysophosphatidic acid but enhances diet-induced adiposity [39], while recent clinical data demonstrated that serum ATX levels are positively associated with metabolic-associated fatty liver disease and hyperuricemia in postmenopausal women [40]. Together, these studies further support the central role of the ATX–LPA pathway in metabolic regulation and liver disease.

Future randomized controlled trials with larger populations, longer follow-up periods, and comprehensive nutritional and molecular assessments are needed to confirm these findings, explore underlying mechanisms, and assess the long-term impact of dietary strategies on the ATX–LPA axis and MASLD progression.

## Figures and Tables

**Table 1:** Demographic and lifestyle characteristics at baseline by sex.

Parameter	All	Sex		
		Female	Male	*p*-Value ^¥^
N	44	22	22	
Age * (years)	46.5 (10.4)	45.4 (11.0)	47.3 (9.5)	
Smoking habit (%)				
Never	39 (95)	18 (90)	21 (100)	0.140
Current	2 (55)	2 (10)	0 (0)	
Adherence to diet—Med (%)				
Low	5 (11)	2 (9)	3 (14)	0.810
Moderate	28 (64)	15 (68)	13 (59)	
High	11 (25)	5 (23)	6 (27)	
Physical activity (%)				
<30 min	8 (20)	4 (20)	4 (19)	0.940
>30 min	33 (80)	16 (80)	17 (81)	
Education (%)				
Secondary School	11 (27)	7 (35)	4 (19)	0.250
High School	22 (54)	11 (55)	11 (52)	
Graduate	8 (20)	2 (10)	6 (29)	

* Mean (SD). ^¥^ χ^2^ test.

**Table 2:** Description of the whole sample: Pre and Post-vegetable-enriched diet.

Parameters	Pre-Diet	Post-Diet	*p*-Value ^¥^
N	44	44	
	Mean (SD)	Mean (SD)	
**Outcome Variable:**			
ATX (ng/mL)	206.28 (52.79)	191.71 (45.73)	<0.001
**Molecule**			
LPA (µg/mL)	11.98 (2.75)	11.72 (3.01)	0.264
PAI-1 (ng/mL)	3.58 (0.90)	3.35 (0.80)	0.220
**Ultrasonographic measures of liver steatosis and fibrosis**			
CAP (dB/m)	313.8 (47.0)	278.5 (48.9)	<0.001
LSM (kPa)	6.6 (3.0)	6.4 (4.1)	0.064
**Anthropometric and clinical parameters**			
SBP (mmHg)	133.1 (13.0)	124.5 (8.6)	0.001
DBP (mmHg)	80.6 (11.4)	76.2 (8.1)	<0.001
PREDIMED questionnaire	8.0 (7.0, 9.0)	11.0 (10.0, 12.0)	<0.001
BMI (kg/m^2^)	36.7 (4.2)	34.8 (4.1)	<0.001
Waist circumference (cm)	113.7 (11.9)	107.4 (12.5)	<0.001
Fat Mass (kg)	40.9 (10.2)	36.0 (9.4)	<0.001
Free Fat Mass (kg)	63.5 (11.8)	62.8 (11.6)	0.125
Body Cell Mass	35.8 (7.8)	35.6 (7.9)	0.338
**Blood Tests:**			
Glucose (mg/dL)	94.5 (8.9)	94.8 (8.4)	0.799
Insulin (µIU/mL)	19.4 (10.4)	15.9 (8.4)	<0.001
Homa IR	4.6 (2.7)	3.8 (2.1)	<0.001
Hemoglobin A1c	5.5 (0.4)	5.4 (0.3)	<0.001
Triglycerides (mg/dL)	123.3 (59.5)	101.5 (54.2)	<0.001
Total Cholesterol (mg/dL)	192.4 (31.4)	177.3 (29.5)	<0.001
HDL Cholesterol (mg/dL)	50.0 (11.4)	47.2 (10.9)	0.008
LDL Cholesterol (mg/dL)	125.8 (28.5)	110.9 (25.2)	<0.001
AST (U/L)	22.2 (10.7)	19.2 (7.7)	<0.001
ALT (U/L)	29.9 (18.1)	22.6 (12.5)	<0.001
γGT (U/L)	24.6 (14.4)	20.2 (12.1)	<0.001
Uric Acid (mg/dL)	5.4 (1.7)	5.4 (1.3)	0.543
Creatinine (mg/dL)	0.9 (0.2)	0.8 (0.2)	0.592
hs-CRP (mg/dL)	0.3 (0.2)	0.4 (0.7)	0.210
WBC (10^6^/µL)	6.36 (1.55)	5.97 (1.42)	0.006
RBC (10^6^/µL)	4.96 (0.44)	4.90 (0.43)	0.249
TSH (µmU/mL)	1.9 (0.9)	1.9 (1.2)	0.083
FT3 (pg/mL)	3.4 (0.5)	3.1 (0.3)	0.002
FT4 (ng/dL)	12.2 (1.2)	11.8 (2.0)	0.423

^¥^ Wilcoxon signed-rank test for paired data. Legend: ATX: Autotaxin; LPA: Lysophosphatidic Acid; PAI1: Plasminogen Activator Inhibitor 1; CAP: Controlled Attenuation Parameter; LSM: Liver Stiffness Measurement; SBP: Systolic Blood Pressure; DBP: Diastolic Blood Pressure; BMI: Body Mass Index; HOMA-IR: Homeostasis Model Assessment for Insulin Resistance; HDL: High-Density Lipoprotein; LDL: Low-Density Lipoprotein; AST: Aspartate Amino Transferase, ALT: Alanine Transaminase; γGT: Gamma-Glutamyl Transpeptidase, hs-CRP: High Sensitivity C-Reactive Protein, WBC: White Blood Cell; RBC: Red Blood Cell; TSH: Thyroid-Stimulating Hormone; FT4: Free Tetraiodothyronine; FT3: Free Triiodothyronine.

**Table 3:** Description of the sample: Sex by Pre- and post-vegetable-enriched diet.

Parameters	Female			Male		
	Pre-Diet	Post-Diet	*p*-Value ^¥^	Pre-Diet	Post-Diet	*p*-Value ^¥^
N	22	22		22	22	
**Outcome Variable:**						
ATX (ng/mL)	234.75 (52.23)	216.79 (43.83)	<0.001	177.82 (35.79)	166.63 (32.31)	0.0075
**Molecule**						
LPA (µg/mL)	11.66 (1.85)	11.03 (2.11)	0.1075	12.30 (3.45)	12.42 (3.62)	0.7691
PAI1 (ng/dL)	3.55 (1.09)	3.36 (0.96)	0.1049	3.60 (0.67)	3.35 (0.63)	0.0049
**Ultrasonographic measures of liver steatosis and fibrosis**						
CAP (dB/m)	291.39 (37.55)	264.06 (32.57)	0.0021	333.10 (46.50)	290.81 (57.37)	<0.001
LSM (kPa)	7.92 (5.84)	6.63 (5.43)	0.0356	6.39 (2.54)	6.13 (1.66)	0.3543
**Anthropometric and clinical parameters**						
SBP(mmHg)	131.10 (12.34)	124.42 (11.30)	0.0363	135.00 (13.58)	124.57 (5.46)	0.0012
DBP (mmHg)	77.95 (11.47)	71.47 (6.80)	0.0006	83.14 (10.91)	80.57 (6.80)	0.0466
PREDIMED questionnaire ^§^	8.50 (7.00, 9.00)	10.5 (10.0, 11.0)	<0.001	7.0 (7.0, 9.0)	11.0 (10.0, 12.0)	<0.001
BMI (kg/m^2^)	36.60 (4.88)	34.75 (4.55)	<0.001	36.80 (3.65)	34.79 (3.78)	<0.001
Waist circumference (cm)	107.30 (10.40)	100.60 (11.03)	<0.001	119.86 (9.97)	113.86 (10.34)	<0.001
Fat Mass (kg)	42.70 (11.32)	37.93 (10.08)	<0.001	39.09 (8.82)	34.15 (8.46)	<0.001
Free Fat Mass (kg)	53.02 (4.65)	52.86 (4.30)	0.3550	73.41 (6.56)	72.22 (7.56)	0.0274
Body Cell Mass	28.76 (2.96)	28.52 (2.95)	0.2382	42.57 (3.97)	42.30 (4.40)	0.2308
**Blood Tests:**						
Glucose (mg/dL)	93.09 (9.06)	94.39 (8.48)	0.7717	96.00 (8.73)	95.23 (8.51)	0.2807
Insulin (µIU/mL)	16.34 (8.60)	13.25 (5.65)	0.0114	22.55 (11.23)	18.55 (9.88)	0.0115
Homa IR	3.81 (2.21)	3.13 (1.45)	0.0234	5.43 (2.94)	4.40 (2.48)	0.0135
Hemoglobin A1c	5.52 (0.42)	5.40 (0.37)	0.0042	5.44 (0.33)	5.30 (0.28)	0.0065
Triglycerides (mg/dL)	118.49 (56.70)	80.78 (24.61)	0.0008	128.05 (63.11)	122.27 (67.07)	0.2625
Total Cholesterol (mg/dL)	190.13 (31.15)	172.16 (32.29)	0.0027	194.64 (32.20)	182.52 (26.19)	0.0053
HDL Cholesterol (mg/dL)	54.24 (12.82)	52.61 (11.67)	0.1082	45.68 (8.00)	41.87 (6.87)	0.0048
LDL Cholesterol (mg/dL)	118.48 (28.32)	105.72 (24.66)	0.0105	132.78 (27.46)	115.93 (25.28)	0.0006
AST (U/L)	20.40 (12.78)	19.37 (10.38)	0.1970	23.95 (8.03)	19.12 (3.79)	0.0011
ALT (U/L)	22.62 (15.57)	19.13 (13.11)	0.0023	37.18 (17.71)	26.08 (11.07)	0.0001
γGT (U/L)	20.14 (14.30)	18.36 (14.65)	0.1016	29.00 (13.33)	22.09 (8.85)	0.0001
Uric Acid (mg/dL)	4.27 (0.94)	4.41 (0.69)	0.7035	6.43 (1.67)	6.31 (1.00)	0.3846
Creatinine (mg/dL)	0.72 (0.10)	0.73 (0.10)	0.6975	1.00 (0.12)	0.96 (0.12)	0.1250
hs-CRP (mg/dL)	0.32 (0.24)	0.40 (0.72)	0.3025	0.25 (0.22)	0.39 (0.68)	0.7035
WBC (10^6^/µL)	5.73 (1.04)	5.52 (1.17)	0.1531	7.04 (1.70)	6.55 (1.61)	0.0086
RBC (10^6^/µL)	4.67 (0.34)	4.66 (0.34)	0.4376	5.25 (0.32)	5.14 (0.37)	0.1511
TSH (µmU/mL)	1.88 (0.87)	1.80 (0.75)	0.3728	1.97 (1.00)	1.94 (1.53)	0.4746
FT3 (pg/mL)	3.22 (0.54)	3.13 (0.35)	0.2471	3.61 (0.39)	3.15 (0.32)	0.0001
FT4 (ng/dL)	11.72 (1.17)	11.44 (2.56)	0.3204	12.71 (1.05)	12.22 (1.30)	0.0864

^¥^ Wilcoxon signed-rank test by paired data. ^§^ Median (IQR). Legend: ATX: Autotaxin; LPA: Lysophosphatidic Acid; PAI1: Plasminogen Activator Inhibitor 1; CAP: Controlled Attenuation Parameter; LSM: Liver Stiffness Measurement; SBP: Systolic Blood Pressure; DBP: Diastolic Blood Pressure; BMI: Body Mass Index; HOMA-IR: Homeostasis Model Assessment for Insulin Resistance; HDL: High-Density Lipoprotein; LDL: Low-Density Lipoprotein; AST: Aspartate Amino Transferase, ALT: Alanine Transaminase; γGT: Gamma-Glutamyl Transpeptidase, hs-CRP: High Sensitivity C-Reactive Protein, WBC: White Blood Cell; RBC: Red Blood Cell, MCV: Mean Corpuscular Volume; TSH: Thyroid-Stimulating Hormone; FT4: Free Tetraiodothyronine; FT3: Free Triiodothyronine.

**Table 4:** Description of the sample: pre- and post-vegetable-enriched diet by sex.

Parameters	Pre-Diet			Post-Diet		
	Female	Male	*p*-Value ^¥^	Female	Male	*p*-Value ^¥^
N	22	22		22	22	
**Outcome Variable:**						
ATX (ng/mL)	234.75 (52.23)	177.82 (35.79)	<0.001	216.79 (43.83)	166.63 (32.31)	<0.001
**Molecule**						
LPA (µg/mL)	11.66 (1.85)	12.30 (3.45)	0.450	11.03 (2.11)	12.42 (3.62)	0.130
PAI1 (ng/dL)	3.55 (1.09)	3.60 (0.67)	0.850	3.36 (0.96)	3.35 (0.63)	0.990
CAP (dB/m)	291.39 (37.55)	333.10 (46.50)	0.004	264.06 (32.57)	290.81 (57.37)	0.088
LSM (kPa)	7.92 (5.84)	6.39 (2.54)	0.280	6.63 (5.43)	6.13 (1.66)	0.690
SBP(mmHg)	131.10 (12.34)	135.00 (13.58)	0.340	124.42 (11.30)	124.57 (5.46)	0.960
DBP (mmHg)	77.95 (11.47)	83.14 (10.91)	0.150	71.47 (6.80)	80.57 (6.80)	<0.001
PREDIMED questionnaire ^§^	8.50 (7.00, 9.00)	7.0 (7.0, 9.0)	0.560	10.5 (10.0, 11.0)	11.0 (10.0, 12.0)	0.220
BMI (kg/m^2^)	36.60 (4.88)	36.80 (3.65)	0.890	34.75 (4.55)	34.79 (3.78)	0.970
Waist circumference (cm)	107.30 (10.40)	119.86 (9.97)	<0.001	100.60 (11.03)	113.86 (10.34)	<0.001
Fat Mass (kg)	42.70 (11.32)	39.09 (8.82)	0.260	37.93 (10.08)	34.15 (8.46)	0.200
Free Fat Mass (kg)	53.02 (4.65)	73.41 (6.56)	<0.001	52.86 (4.30)	72.22 (7.56)	<0.001
Body Cell Mass	28.76 (2.96)	42.57 (3.97)	<0.001	28.52 (2.95)	42.30 (4.40)	<0.001
**Blood Tests:**						
Glucose (mg/dL)	93.09 (9.06)	96.00 (8.73)	0.280	94.39 (8.48)	95.23 (8.51)	0.740
Insulin (µIU/mL)	16.34 (8.60)	22.55 (11.23)	0.046	13.25 (5.65)	18.55 (9.88)	0.035
Homa IR	3.81 (2.21)	5.43 (2.94)	0.045	3.13 (1.45)	4.40 (2.48)	0.043
Hemoglobin A1c	5.52 (0.42)	5.44 (0.33)	0.500	5.40 (0.37)	5.30 (0.28)	0.320
Triglycerides (mg/dL)	118.49 (56.70)	128.05 (63.11)	0.600	80.78 (24.61)	122.27 (67.07)	0.009
Total Cholesterol (mg/dL)	190.13 (31.15)	194.64 (32.20)	0.640	172.16 (32.29)	182.52 (26.19)	0.250
HDL Cholesterol (mg/dL)	54.24 (12.82)	45.68 (8.00)	0.011	52.61 (11.67)	41.87 (6.87)	<0.001
LDL Cholesterol (mg/dL)	118.48 (28.32)	132.78 (27.46)	0.110	105.72 (24.66)	115.93 (25.28)	0.200
AST (U/L)	20.40 (12.78)	23.95 (8.03)	0.270	19.37 (10.38)	19.12 (3.79)	0.920
ALT (U/L)	22.62 (15.57)	37.18 (17.71)	0.006	19.13 (13.11)	26.08 (11.07)	0.064
γGT (U/L)	20.14 (14.30)	29.00 (13.33)	0.039	18.36 (14.65)	22.09 (8.85)	0.310
Uric Acid (mg/dL)	4.27 (0.94)	6.43 (1.67)	<0.001	4.41 (0.69)	6.31 (1.00)	<0.001
Creatinine (mg/dL)	0.72 (0.10)	1.00 (0.12)	<0.001	0.73 (0.10)	0.96 (0.12)	<0.001
hs-CRP (mg/dL)	0.32 (0.24)	0.25 (0.22)	0.390	0.40 (0.72)	0.39 (0.68)	0.950
WBC (10^6^/µL)	5.73 (1.04)	7.04 (1.70)	0.003	5.52 (1.17)	6.55 (1.61)	0.020
RBC (10^6^/µL)	4.67 (0.34)	5.25 (0.32)	<0.001	4.66 (0.34)	5.14 (0.37)	<0.001
TSH (µmU/mL)	1.88 (0.87)	1.97 (1.00)	0.770	1.80 (0.75)	1.94 (1.53)	0.710
FT3 (pg/mL)	3.22 (0.54)	3.61 (0.39)	0.009	3.13 (0.35)	3.15 (0.32)	0.800
FT4 (ng/dL)	11.72 (1.17)	12.71 (1.05)	0.005	11.44 (2.56)	12.22 (1.30)	0.210

^¥^ Wilcoxon signed-rank test by paired data. ^§^ Median (IQR). Legend: ATX: Autotaxin; LPA: Lysophosphatidic Acid; PAI1: Plasminogen Activator Inhibitor 1; CAP: Controlled Attenuation Parameter; LSM: Liver Stiffness Measurement; SBP: Systolic Blood Pressure; DBP: Diastolic Blood Pressure; BMI: Body Mass Index; HOMA-IR: Homeostasis Model Assessment for Insulin Resistance; HDL: High-Density Lipoprotein; LDL: Low-Density Lipoprotein; AST: Aspartate Amino Transferase, ALT: Alanine Transaminase; γGT: Gamma-Glutamyl Transpeptidase, hs-CRP: High Sensitivity C-Reactive Protein, WBC: White Blood Cell; RBC: Red Blood Cell, MCV: Mean Corpuscular Volume; TSH: Thyroid-Stimulating Hormone; FT4: Free Tetraiodothyronine; FT3: Free Triiodothyronine.

**Table 5:** Generalized Estimating Equation (GEE): expected values ATX by time (Pre and Post vegetable-enriched Diet).

ATX (ng/mL)	β	*p*-Value	95%CI
Model *a*:			
Pre Diet	0.00		
Post Diet	−13.80	<0.001	−18.04; −9.56
Model *b*:			
Pre Diet	0.00		
Post Diet	−12.44	<0.001	−17.40; −7.48
Model *c*:			
Pre Diet	0.00		
Post Diet	−9.87	<0.001	−15.29; −4.46
LSM	2.25	0.009	0.57; 3.93

Model *a*: univariate. Model *b*: adj for sex (Female vs. Male) and Age, Model *c*: adj for sex (Female vs. Male), Age50 (<50 vs. ≥50 yrs), LPA, FFM, PAI1, Hemoglobin A1c. ATX: Autotaxin; LSM: Liver Stiffness Measurement; LPA: Lysophosphatidic Acid; PAI1: Plasminogen Activator Inhibitor 1; FFM: Fat-Free Mass; β: regression coefficient; CI: Confidence Interval.

**Table 6:** Generalized Estimating Equation (GEE): expected values ATX by time (Pre and Post vegetable-enriched Diet) and sex.

ATX (ng/mL)		Female			Male	
	β	*p*-Value	95%CI	β	*p*-Value	95%CI
Model *d*:						
Pre Diet	0.00			0.00		
Post Diet	−17.96	<0.001	−25.36; −10.55	−9.68	0.002	−15.67; −3.69
Model *e*:						
Pre Diet	0.00					
Post Diet	−12.24	0.005	−20.85; −3.62	−9.43	0.014	−16.94; −1.92
LSM	2.18	0.049	0.00; 4.35	2.61	0.078	−0.29; 5.52

Model *d*: univariate. Model *e*: adj for LSM, Age50 (<50 vs. ≥50 yrs), FFM, PAI1, LPA, Hemoglobin A1c. ATX: Autotaxin; LSM: Liver Stiffness Measurement; LPA: Lysophosphatidic Acid; PAI1: Plasminogen Activator Inhibitor 1; FFM: Fat-Free Mass; β: regression coefficient; CI: Confidence Interval.

## Data Availability

The datasets generated and/or analyzed during the current study are available in the Figshare repository, [DOI: 10.6084/m9.figshare.30095326].

## Abbreviations

The following abbreviations are used in this manuscript:

MASLD	Metabolic Dysfunction-Associated Steatotic Liver Disease
MASH	Metabolic Steatohepatitis
CVD	Cardiovascular Diseases
ATX	Autotaxin
LPA	Lysophosphatidic Acid
BMI	Body Mass Index
WC	Waist Circumference
CAP	Controlled Attenuation Parameter
LSM	Liver Stiffness Measurement
FSG	Fasting Serum Glucose
LDL-C	Low-Density Lipoprotein Cholesterol
HDL-C	High-Density Lipoprotein Cholesterol
AST	Aspartate-Amino transferase
ALT	Alanine-Amino transferase
γGT	Gamma Glutaminyl-Transferase
WBC	White Blood Cell
RBC	Red Blood Cell
TSH	Thyroid-Stimulating Hormone
FT4	Free Tetraiodothyronine
FT3	Free Triiodothyronine
HbA1c	Glycated hemoglobin
HOMA-IR	Homeostasis Model Assessment of Insulin Resistance
IPAQ	International Physical Activity Questionnaire
PREDIMED	Prevention with Mediterranean Diet
FM	Fat Mass
FFM	Fat-Free Mass
BMC	Body Cell Mass
TBW	Total Body Water
